# Diffusion tensor imaging quantifying the severity of chronic hepatitis in rats

**DOI:** 10.1186/s12880-020-00466-3

**Published:** 2020-07-02

**Authors:** Mengping Huang, Xin Lu, Xiaofeng Wang, Jian Shu

**Affiliations:** Department of Radiology, The Affiliated Hospital of Southwest Medical University, 25 Taiping Street, Luzhou, Sichuan 646000 People’s Republic of China

**Keywords:** Chronic hepatitis, Liver fibrosis, Diffusion tensor imaging, Average diffusion coefficient, Fractional anisotropy

## Abstract

**Background:**

Diffusion tensor imaging (DTI) is mainly used for detecting white matter fiber in the brain. DTI was applied to assess fiber in liver disorders in previous studies. However, the data obtained have been insufficient in determining if DTI can be used to exactly stage chronic hepatitis. This study assessed the value of DTI for staging of liver fibrosis (F), necroinflammatory activity (A) and steatosis (S) with chronic hepatitis in rats.

**Methods:**

Seventy male Sprague-Dawley rats were divided into a control group(*n* = 10) and an experimental group(*n* = 60). The rat models of chronic hepatitis were established by abdominal subcutaneous injections of 40% CCl_4_. All of the rats underwent 3.0 T MRI. Regions of interest (ROIs) were subjected to DTI to estimate the MR parameters (rADC value and FA value). Histopathology was used as the reference standard. Multiple linear regression was used to analyze the associations between the MR parameters and pathology. The differences in the MR parameters among the pathological stages were evaluated by MANOVA or ANOVA. The LSD test was used to test for differences between each pair of groups. ROC analysis was also performed.

**Results:**

The count of each pathology was as follows: F0(*n* = 15), F1(*n* = 11), F2(*n* = 6), F3(*n* = 9), F4(n = 6); A0(*n* = 8), A1(*n* = 16), A2(n = 16), A3(*n* = 7); S0(*n* = 10), S1(n = 7), S2(*n* = 3), S3(n = 11), S4(n = 16). The rADC value had a negative correlation with liver fibrosis (*r* = − 0.392, *P* = 0.008) and inflammation (*r* = − 0.359, *P* = 0.015). The FA value had a positive correlation with fibrosis (*r* = 0.409, *P* = 0.005). Significant differences were found in the FA values between F4 and F0 ~ F3 (*P* = 0.03), while no significant differences among F0 ~ F3 were found (*P* > 0.05). The AUC of the FA value differentiating F4 from F0 ~ F3 was 0.909 (*p* < 0.001) with an 83.3% sensitivity and an 85.4% specificity when the FA value was at the cut-off of 588.089 (× 10^− 6^ mm^2^/s).

**Conclusion:**

The FA value for DTI can distinguish early cirrhosis from normal, mild and moderate liver fibrosis, but the rADC value lacked the ability to differentiate among the fibrotic grades. Both the FA and rADC values were unable to discriminate the stages of necroinflammatory activity and steatosis.

## Background

Chronic hepatitis is a typical chronic diffuse liver disease that can be caused by many factors [1]. The basic pathological changes associated with chronic hepatitis include hepatic inflammation, liver fibrosis and fatty infiltration and the disease can further develop into cirrhosis and even liver cancer and liver failure [2, 3]. Studies [4] have shown that early chronic hepatitis manifests as a dynamic and reversible lesion. Furthermore, early diagnosis and accurate staging of chronic hepatitis are clinically significant in evaluating the severity and progress of the disease. Percutaneous liver biopsy is considered the gold standard, but patients often reject this invasive technique, as they usually have no symptoms [5].

Several noninvasive methods have been presented chronic hepatitis, among which the most promising ones are ultrasound elastography [6] and magnetic resonance elastography [7]; however, the former is inadequate or unavailable in obese and abdominal dropsy patients and the latter is expensive. DTI is a mature magnetic resonance imaging (MRI) sequence developed on the basis of diffusion weighted imaging (DWI). Compared with unidirectional or three orthogonal directional DWI, DTI quantifies the diffusivity of water molecules by using six or more different directions of diffusion sensitive gradients, traces the fiber bundle shape, and visually reveals the microstructural characteristics of biological tissues. The DTI sequence produces an average diffusion coefficient (rADC) image, a fractional anisotropy (FA) image, a relative anisotropy image and the corresponding values (among which the rADC and FA values are widely used) [8].

DTI is mainly used to detect white matter fiber in the nervous system [9]. Some studies [10–13] have applied DTI for chronic hepatitis because fibrosis always emerges in liver damage. Previous studies [12, 13] have shown that the CCl_4_-induced liver fibrosis animal model is a mature technique and can mirror the pathophysiologic processes of fibrogenesis in humans [14]. However, insufficient data have been obtained. Our study assessed whether the rADC and FA values of DTI can help to distinguish the different stages of liver fibrosis, necroinflammatory activity and steatosis in rats with chronic hepatitis induced by abdominal subcutaneous injection of CCl_4._

## Methods

### Establishment of a chronic hepatitis model in rats

This study was approved by the Animal Ethics Committee of the Southwest Medical University. Seventy 7-week-old male Sprague-Dawley rats weighing 150–200 g were randomly divided into a control group (*n* = 10) and an experimental group(*n* = 60). The rats were purchased from Animal Experimental Center of Southwest Medical University. A chronic hepatitis model was induced in rats by abdominal subcutaneous injection of a 40%CCl_4_ suspension (99.9% carbon tetrachloride: vegetable oil = 4:6) at a dose of 0.3 ml/100 g twice a week. The rats in the control group were injected with 0.9% sodium chloride at the same dose and in the same way. The animals were raised under standard conditions and had free access to food and water.

### MR imaging

Five weeks after injecting CCl_4_, 6 to 10 rats for the test group and 1or 2 rats for the control group were randomly selected for an MRI scan every week. The rats were supinely fixed on boards under anesthesia during scanning by intraperitoneal injection of 1% pentobarbital sodium at a dose of 0.5 ml/100 g.

The MR exams were performed on a 3.0 T MRI scanner(Achieva 3.0 T, Philips, Netherlands) using 8-channel knee coils. Spin-echo echo-planar imaging diffusion tensor imaging sequence; two b values (0 and 800 s/mm^2^); 15 diffusion gradient directions; TR, 3907 ms; TE, 86 ms; FOV, 100 mm × 100 mm, thickness, 2.0 mm; NSA, 3; matrix, 240.

### Image analysis

**Fig. 1:**
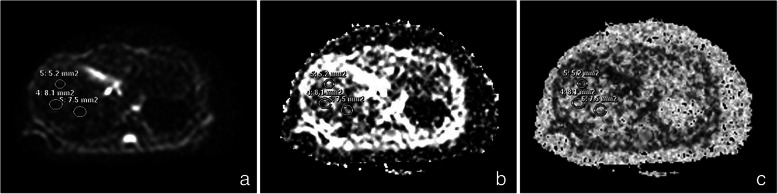
rADC and FA value calculation. **a** The three circular ROIs ranging from 5 mm^2^ ~ 10 mm^2^ were placed on the DTI images at b = 0 s/mm^2^. **b** ROIs were copied to the rADC image, and the computer calculated the rADC values. **c** ROIs were copied to the FA image, and the computer calculated the FA values

Two radiologists who were blinded to the pathological results used a postprocessing workstation (Philips Extended MR WorkSpace 2.6.3.4) to generate functional imaging maps (rADC image and FA image) and measure the quantitative indicators of the regions of interest (ROIs). Three circular ROIs per slice ranging from 5 mm^2^ ~ 10 mm^2^ were placed on two consecutive slices of the DTI images of b = 0 s/mm^2^ and were then copied to the same slices of rADC images and FA images (Fig.1). The mean values of the six ROIs measured by the two radiologists were estimated. The average value of the two radiologists’ measurements for each specimen was utilized as the final measurement. Care was taken to avoid large vessels, and the edge of the ROI was at least 3 mm away from the border of the liver.

### Histopathological evaluation

For the pathological evaluation, the rats were sacrificed by cervical dislocation immediately after the MRI scan. In addition, hematoxylin and eosin staining and Masson staining were performed. According to the METAVIR scoring system [15], hepatic fibrosis (F) was classified on a 5-point scale (F0: no fibrosis; F1: portal fibrosis without septa; F2: portal fibrosis with rare septa; F3: numerous septa without cirrhosis; F4: cirrhosis) and necroinflammatory activity (A) was classified on a 4-point scale(A0: no activity; A1: mild activity; A2: moderate activity; A3: severe activity). Steatosis(S) is depended on the percentage of liver cells containing fat droplets as follows: S0(0–5%), S1 (6–30%), S2 (31–50%), S3 (51–75%), and S4 (> 75%). The liver sections were assessed by two pathologists who did not know the radiologic outcome.

### Statistical analysis

The intraclass correlation coefficient (ICC) was calculated to evaluate the reliability of the values measured by the two radiologists. Multiple linear regression (Enter model) analysis was used to examine the relationships between the MR parameters (rADC value and FA value) and pathological stage (necroinflammation, hepatic fibrosis and steatosis). The differences in the magnetic resonance parameters among the pathological stages were evaluated by a multi factor analysis of variance (MANOVA) or a one-way analysis of variance (ANOVA). The least significant difference (LSD) method was used to test the differences between each pair of groups. To evaluate the diagnostic performance of the FA value for the assessment of fibrosis stage, a receiver operating characteristic (ROC) curve was constructed. A *P* value less than 0.05 was considered statistically significant.

## Result

### General feature of the animal models and pathologic results

Sixteen rats died in the experimental group during induction of the model. Five specimens in the experimental group and one specimen in the control group which had a low signal-to-noise ratio were eliminated despite taking measures to reduce artifacts. Finally, there were 47 specimens available including 8(8/10) control group rats and 39(39/60) chronic hepatitis rats. The statistical data were as follows: F0(*n* = 15), F1(*n* = 11), F2(*n* = 6), F3(*n* = 9), F4(n = 6); A0(*n* = 8), A1(*n* = 16), A2(n = 16), A3(*n* = 7); S0(*n* = 10), S1(n = 7), S2(*n* = 3), S3(n = 11), S4(n = 16). All the data are summarized in Table 1.

**Table 1:** Distribution of rADC value and FA value in pathology of the liver

Pathological stage	number	rADC (× 10^− 6^ mm^2^/s)	FA (× 10^− 6^ mm^2^/s)
A0F0S0	8	923.008 ± 69.899	505.421 ± 34.550
A1F0S1	2	885.900 ± 19.351	489.075 ± 44.182
A1F0S4	1	1167.383	372.433
A1F1S1	2	941.417 ± 128.057	455.658 ± 118.688
A1F1S4	4	852.388 ± 150.181	553.717 ± 99.250
A1F2S0	1	875.067	582.250
A1F2S3	2	760.892 ± 30.205	526.508 ± 55.425
A1F2S4	2	839.900 ± 47.588	526.508 ± 55.425
A1F3S3	2	774.908 ± 88.565	535.308 ± 140.184
A2F0S4	1	852.233	405.000
A2F1S3	1	683.100	664.783
A2F1S4	3	795.483 ± 128.615	465.306 ± 181.995
A2F3S1	1	637.583	598.350
A2F3S2	2	818.750 ± 26.729	494.017 ± 73.044
A2F3S3	1	776.233	536.083
A2F3S4	1	866.550	582.167
A2F4S0	1	743.350	634.433
A2F4S1	2	651.425 ± 28.555	731.483 ± 21.920
A2F4S2	1	701.783	759.617
A2F4S3	2	774.775 ± 94.599	576.917 ± 26.328
A3F0S3	2	774.975 ± 63.062	530.283 ± 58.266
A3F0S4	1	763.033	606.250
A3F1S4	1	826.183	525.267
A3F2S4	1	736.933	544.250
A3F3S3	1	804.083	545.117
A3F3S4	1	803.050	566.138

Abbreviations: A, necroinflammatory activity; *F* fibrosis, *S* steatosis, *rADC* average diffusion coefficient, *FA* fractional anisotropy

### MRI quantitative indicators

The ICC of the rADC value was 0.852 (*P*<0.001), and that of the FA value was 0.922 (*P*<0.001). High measurement repeatability between the two observers indicated the clinical feasibility of this method.

The results of the multiple linear regression analysis are presented in Table 2. The rADC value was correlated with fibrosis (*r* = − 0.392, *P* = 0.008) and necroinflammatory activity (*r* = − 0.359, *P* = 0.015), but not with steatosis (*P* = 0.452). The FA value was related to the degree of fibrosis (*r* = 0.409, *P* = 0.005), but not to inflammatory activity(*P* = 0.236) or steatosis (*P* = 0.115). Table 3 shows the rADC value and FA value of the different stages of liver fibrosis and indicates that the rADC value decreased with the severity of liver fibrosis while the FA value increased.

**Table 2:** Results of the regression analysis between quantitative indexes of DTI and pathology

Pathologic staging	rADC value			FA value		
	*B*	*r*	*P*	*B*	*r*	*P*
A	−48.243	−0.359	0.015^*^	20.924	0.180	0.236
F	−29.365	−0.392	0.008^*^	28.175	0.409	0.005^*^
S	7.952	0.115	0.452	−15.368	−0.238	0.115

Abbreviations: A, necroinflammatory activity; *F* fibrosis, *S* steatosis, *rADC* average diffusion coefficient, *FA* fractional anisotropy

*Significant at *P* < 0.05. *B* value, unstandardized coefficient. *r* value, partial correlation coefficient

**Table 3:** Results of the quantitative analysis of rADC and FA value in according to the fibrotic stage

Fibrotic stage	rADC value(×10^−6^ mm^2^/s)	FA value(×10^−6^ mm^2^/s)
F0	899.231 ± 109.210	497.718 ± 61.249
F1	835.283 ± 129.938	519.286 ± 123.599
F2	802.264 ± 61.647	531.164 ± 52.087
F3	786.091 ± 70.868	542.944 ± 66.228
F4	716.256 ± 72.060	668.475 ± 84.078

Abbreviations: *F* fibrosis, *rADC* average diffusion coefficient, *FA* fractional anisotropy

**Fig. 2:**
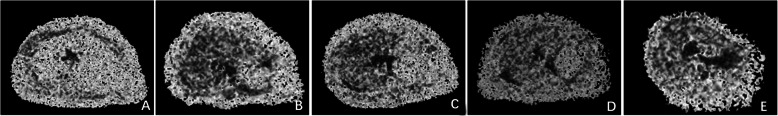
FA images of the different liver fibrosis stages in rats. A ~ E represent F0, F1, F2, F3 and F4, respectively

Based on the means from MANOVA, no significant differences were found among the stages of fibrosis (*F* = 1.250, *P* = 0.309) or inflammatory activity (*F* = 1.487, *P* = 0.236) for the rADC value. When analyzed by ANOVA, the FA values among the different fibrosis groups were significantly different (*F* = 4.750, *P* = 0.03). There was a significant difference in the FA value between F4 and F0 ~ F3 (*P* < 0.05), while no significant differences among F0 ~ F3 were found (*P* > 0.05). The area under the ROC curve (AUC) of the FA value that differentiated F4 from F0 ~ F3 was 0.909(*p* < 0.001) at a cut-off of 588.089(× 10^− 6^ mm^2^/s), with an 83.3% sensitivity and an 85.4% specificity. Figure 2 shows FA images of the different liver fibrosis stages.

## Discussion

Our study indicated that the rADC value was negatively related to hepatic fibrosis and necroinflammatory activity but not to steatosis. However, the rADC value was not significantly different among the stages of fibrosis or necroinflammatory activity. The FA value had a positive correlation with the degree of fibrosis, but no correlation with necroinflammatory activity or steatosis. The FA value of F4 was significantly different from those of F0 ~ F3, while there were no significant differences among F0 ~ F3, which suggests that the liver FA value can distinguish early cirrhosis(F4) but it has little significance in differentiating normal, mild and moderate liver fibrosis(F0 ~ F3).

Chronic hepatitis is characterized by a series of histological features including hepatic inflammation, liver fibrosis and fatty infiltration. As a consequence of chronic injury, the inflammatory system is first activated, which includes the activation of resident innate inflammatory cells and the recruitment of additional inflammatory cells. Then, hepatic stellate cells are activated and transformed into fibroblasts, which produce a great quantity of collagen in the extracellular matrix (ECM). The excessive deposition of ECM finally results in abnormal changes in hepatic structure and hemodynamics inside and outside of the liver [3]. When pseudolobules and nodules form, cirrhosis of the liver develops. The aggravation of hepatic inflammation is the basis of fibrosis development and the proliferation of intrahepatic fibrous septa can lead to the aggravation of hepatic inflammatory necrosis. Meanwhile, liver steatosis is also a risk factor for the progression of fibrosis. However, these abnormalities always display an otherwise normal morphology and signal in conventional MRI. With the emergence of functional MRI, the focus has changed from morphology to function [16]. For example, a gadoxetic acid enhanced MRI was used to assess liver function in cirrhosis [17]. DTI is one of the functional MRIs that can effectively detect the free diffusion rate of water molecules with different structures in vivo and can more accurately reflect changes in the direction of water molecule dispersion, which provides both functional and microstructural information for the liver through water diffusivity and diffusion anisotropy quantitation and may contribute to the evaluation of liver fibrosis [13].

The reduction in the rADC value with fibrosis observed in our study was in accordance with the findings of most prior research [18–20]. However, both our study and previous studies [11, 19] revealed that the rADC value lacked the ability to differentiating the fibrotic grades. The relationship between the FA value and liver fibrosis and the evaluation of the FA value for fibrosis staging differ. Cheung et al. [13] found that the FA value of rats 2 weeks after CCl_4_ insult was significantly lower than that before and 4 weeks after the insult, while the FA value at 4 weeks after the CCl_4_ insult was not significantly different from that before insult, which suggests that FA value can reveal the progression of liver fibrosis, especially early cirrhosis. Other animal research using C57BL/6 mice [12] reported that FA was negatively correlated with hepatic fibrosis and the model group(*n* = 20) had a lower FA than the control group(*n* = 16). However, the sample size in this study was relatively small (F1 = 4, F2 = 11, F3 = 5), and missing value occurred for F4. In contrast, Tosun M et al. [11] found that the FA values showed a trend toward higher values with an increasing fibrotic stage, but there were no statistically significant differences between the FA values at the different fibrotic stages. Our study also found a positive correlation between the FA value and the fibrosis degree. Meanwhile, our study showed that the FA value of F4(early cirrhosis) was significantly different from that of F0 ~ F3. Liver cirrhosis is the end stage of liver fibrosis which has a small chance of reversal and a high risk of developing into complications and hepatocellular carcinoma. However, radiologists cannot diagnose early cirrhosis by relying on conventional medical imaging because the morphological changes are not obvious. Our study found that the FA value of DTI can distinguish early cirrhosis, which may help physicians take early measures. One explanation for our study results is that with an increasing degree of liver fibrosis, the free movement of water molecules in the liver is affected by the presence of the fibers, and the movement direction tends to be consistent or opposite, which increases the FA value. The FA values of F0-F3 were not significantly different because in the early stages of liver fibrosis, the distribution of collagen fibers is not regular and directional, which results in the restricted diffusion of water molecules in all directions and leads to a less obvious direction of the main axis of water molecule movements. As a result, the FA value did not markedly change. With the progress of fibrosis, the fibrous bundles increased, joined into strips, flaked, and rearranged, which made the main axis of water molecule diffusion more obvious, causing the FA value to increase significantly in F4(early cirrhosis).

There are limited data regarding the relationship between liver necroinflammatory grade and DTI measurements. In general, studies [10, 11] found that liver ADC values were inversely correlated with inflammation. However, he rADC cannot discriminate between the different inflammation grades. Both our study and Tosun M’s [11] study demonstrated that the FA value was not related to inflammation grade. The explanation for the rADC decrease with increasing inflammation grades may be the large number of inflammatory cells and factors that helped restrict the free movement ability of water molecules. However, it did not influence the movement direction of the water molecules, therefore, the FA value had no means with inflammation.

Because clinical therapy depends on the fibrotic stage and inflammatory grade, prior studies mainly used DTI for liver fibrosis and inflammation detection [10, 11]. However, some scholars emphasized that the ADC [21–24] and FA value [12] in the liver need to be carefully interpreted in the presence of hepatic steatosis. Besheer T [21] demonstrated that hepatic steatosis should always be considered when assessing hepatic fibrosis, and their study revealed that detected hepatic steatosis would underestimate the ADC value in patients with chronic hepatitis C. Accordingly, our study considered steatosis. However, our study results did not show a relationship between steatosis and either the rADC or FA value, which did not agree with the findings of some previous research [21, 24–26]. Other studies [15, 27] demonstrated no significant relationship between the ADC value and steatosis, which was similar to our results. The inconsistent relationship reported by researchers between MR measurements and steatosis cannot be accurately explained. It is possible that different MR machines and parameters or different group standards could affect the results.

There were limitations in our study. First, the main deficiency was that the echo-planar imaging sequence for DTI had a low signal-to-noise ratio and artifactual interference. Second, the distribution of pathological groups was uneven. The rats had individual differences in sensitivity to the induction of chronic hepatitis after injection of the same dose of drugs, which made the sample size of some groups relatively small. Future studies adopting a high signal-to-noise ratio sequence are needed. Chronic liver disease patients who have undergone hepatectomy could enroll in future studies.

## Conclusion

Our experiment showed that the rADC value of the DWI sequence was inversely related to hepatic fibrosis and inflammation and the FA value had a positive correlation with the degree of fibrosis. The FA value had high diagnostic accuracy in differentiating early cirrhosis and thus could be a potential marker for diagnosing liver cirrhosis. However, the rADC value lacked the ability to differentiate fibrotic grades. Overall, neither the FA nor rADC value can determine the stages of necroinflammatory activity and steatosis.

## Data Availability

The datasets used and analyzed during the current study are available from the corresponding author on reasonable request at shujiannc@163.com.

## Abbreviations

DTI: Diffusion tensor imaging
F: Liver fibrosis
A: Necroinflammatory activity
S: Steatosis
MRI: Magnetic resonance imaging
DWI: Diffusion weighted imaging
rADC: Average diffusion coefficient
FA: Fractional anisotropy
ROIs: Region of interests
ICC: Intraclass correlation coefficient
MANOVA: Multi factor analysis of variance
ANOVA: One-way analysis of variance
LSD: Least significant difference
ROC: Receiver operating characteristic curve
